# Female pond bats hunt in other areas than males and consume lighter prey when pregnant

**DOI:** 10.1093/jmammal/gyad096

**Published:** 2023-10-16

**Authors:** Anne-Jifke Haarsma, Eelke Jongejans, Elza Duijm, Carolien van der Graaf, Youri Lammers, Milan Sharma, Henk Siepel, Barbara Gravendeel

**Affiliations:** Radboud University, Radboud Institute for Biological and Environmental Sciences, Heyendaalseweg 135, 6525 AJ, Nijmegen, The Netherlands; Naturalis Biodiversity Center, Evolutionary Ecology Group, Darwinweg 2, 2333 CR Leiden, The Netherlands; Radboud University, Radboud Institute for Biological and Environmental Sciences, Heyendaalseweg 135, 6525 AJ, Nijmegen, The Netherlands; Netherlands Institute of Ecology, Department of Animal Ecology, Droevendaalsesteeg 10, 6708 PB Wageningen, The Netherlands; Naturalis Biodiversity Center, Evolutionary Ecology Group, Darwinweg 2, 2333 CR Leiden, The Netherlands; Bat Research Consultancy Vroegvlieger, Ellekomstraat 70, 2573 XG Den Haag, The Netherlands; Naturalis Biodiversity Center, Evolutionary Ecology Group, Darwinweg 2, 2333 CR Leiden, The Netherlands; Naturalis Biodiversity Center, Evolutionary Ecology Group, Darwinweg 2, 2333 CR Leiden, The Netherlands; HZ University of Applied Sciences, Life Sciences cluster, Edisonweg 4, 4382 NW Vlissingen, The Netherlands; Radboud University, Radboud Institute for Biological and Environmental Sciences, Heyendaalseweg 135, 6525 AJ, Nijmegen, The Netherlands; Radboud University, Radboud Institute for Biological and Environmental Sciences, Heyendaalseweg 135, 6525 AJ, Nijmegen, The Netherlands; Naturalis Biodiversity Center, Evolutionary Ecology Group, Darwinweg 2, 2333 CR Leiden, The Netherlands

**Keywords:** Chiroptera, environmental DNA (eDNA), high-throughput sequencing, intraspecific competition, metabarcoding, *Myotis dasycneme*

## Abstract

Animals with large energy requirements are forced to optimize their hunting strategy, which may result in differentiation of the diet between sexes and across seasons. Here, we examined spatiotemporal variation in the diet of both sexes of the Pond Bat *Myotis dasycneme*, a species known to have spatial segregation of sexes when the young are born and lactating. Fecal pellets were collected from live animals for a period of 15 years at various locations in the Netherlands. A total of 535 pellets were successfully analyzed by microscopy and an additional 160 pellets by DNA metabarcoding. Morphological and molecular analyses showed that the diet of pregnant and lactating pond bats differed significantly from the diet of females with no reproductive investment. Further analyses of the data showed that pregnant female pond bats are highly dependent on small prey and pupae, mainly nonbiting midges and mosquitoes (Diptera: Chironomidae and Culicidae). These insects can be found in large quantities in peatlands intersected with shallow waterways, the habitat type in which female pond bats were observed more often than males. Our results suggest that during pregnancy the spatial segregation of sexes coincides with sex-specific diets, which might reflect habitat selection based on energy requirements, in addition to lowered intraspecific competition.

## Teaser

When pregnant or lactating, female pond bats occur mainly in peatland with shallow waterways, separate from males. Morphological and molecular analyses of fecal pellets show that this spatial segregation of sexes coincides with sex-specific diets—pregnant females highly depend on small, abundant prey like nonbiting midges and mosquitoes, which might reflect habitat selection based on energy requirements.

In periods of large energy requirements animals are forced to either optimize their hunting strategy by searching for the best hunting areas, and/or optimize energy and nutrient content using other foraging strategies (Loveridge et al. 2009). In field studies of social mammals such as rodents (Schradin et al. 2010), carnivores (Field et al. 2007; Robertson et al. 2014), and primates (Yamagiwa et al. 2003)—where multiple breeding females live in groups—reproductive competition between females is often intense, and groups are often highly territorial and adjust their territory size according to resources. Intraspecific competition can also occur between sexes, potentially resulting in diet differentiation between them (Young et al. 2012; Kernaléguen et al. 2015). This pattern can be expected in mammals, because females that are raising young tend to have much higher energy requirements than males (McHuron et al. 2017), resulting in situations where the females dominate the best hunting areas (Stockley and Bro-Jørgensen 2011; Smith et al. 2018). However, simply observing that males and females occupy different areas during pregnancy and lactation is not sufficient to conclude that this segregation is related to their diet.

Differences in energy requirements among sexes of communal breeding *Myotis* spp. bats result in behavioral adaptations where females arrive earlier (mid-March) in the hunting area, while male bats leave their hibernacula between mid-April and mid-May so that the best areas are taken by the females (Haarsma et al. 2019). Other behavioral adaptations may include aggressive behavior from females toward males in the same hunting area, leading to spatial segregation of the sexes and thus dietary differences (Stevens and Platt 2015). We investigated whether diet differentiation indeed occurs in line with energy requirements differing per habitat, season, and sex in the Pond Bat, *Myotis dasycneme* (Boie 1825). Pond bats are specialized water trawlers, with the ability to capture emerging insects from the water surface with their large hind feet. Water temperature is an important factor influencing the emergence behavior of macrofauna. As a rule, sediment helps to warm water faster, so water in peat habitat, which tends to be “brown,” will be warmer than water in sandy habitats, which tends to be more translucent (Pilla et al. 2018). The same rule can be applied for the depth of a waterway—shallow lakes and ditches (<1.5 m deep) tend to warm faster than deeper water surfaces. These differences in water temperature, and correlated insect yield, will be most apparent during springtime and early summer, and thus play an important role in prey availability during the period of the pregnancy and lactation of pond bats.

Dietary analyses of insectivorous bats have always been hampered by the fact that direct observations of prey consumption are difficult to obtain for these predominantly nocturnally active animals. In addition, many species are legally protected so that invasive sampling is not allowed. Morphological examinations of fecal pellets can provide important first insights into bat diets but are time-consuming and only provide a taxonomic resolution of prey taxa consumed up to insect order, or rarely, family level. However, DNA metabarcoding has revolutionized dietary analyses of mammals (Zeale et al. 2011; Haarsma et al. 2016; Galan et al. 2018). Hundreds of samples can be processed more rapidly and identification up to the species level can be obtained, provided sufficient and relevant reference data are available. However, obtaining reliable results from DNA metabarcoding is not straightforward, and often results in false positives due to contamination, amplification artifacts, and chimeras produced during PCR (Galan et al. 2018). In addition, false negatives can occur due to PCR inhibitors present in feces, low quality of prey DNA, or the lack of prey sequences in the DNA databank. We therefore consider it important to combine metabarcoding with microscopy to cross-check results.

After identifying the typical habitat structures of a female versus male hunting area, we conducted a combined microscopic and metabarcoding study of fecal pellets. These pellets were collected throughout the Netherlands from 857 individual pond bats to answer our main research question: what is the sex-specific diet of pond bats throughout the seasons? First, we hypothesized that female pond bats, because of their higher energy requirements, gather heavier food items than males, and therefore hunt in areas where heavier prey can be expected. Second, we expected the dietary differences between the sexes to be most pronounced during spring and early summer, when females have high energetic costs due to pregnancy and lactation.

## Materials and Methods

### Study species.

Pond bats are colonial animals that roost in buildings. During the breeding season, from early spring until late summer, males and females live in segregated habitats (Haarsma and Tuitert 2009). Only during the mating season from late summer until winter do the sexes meet and use the same roosts. Just like most other species in the genus *Myotis*, pond bats are slightly dimorphic—on average female temperate zone bats are about 20% heavier than males (females 18.5 g and males 16.6 g; Stevens and Platt 2015; Dietz and Kiefer 2016). Pond bats have a fragmented distribution in Europe (Hanák and Gaisler 1965; Horáček and Hanák 1989), ranging from Latvia to the Netherlands, and inhabiting a restricted set of habitats predominantly along rivers and in deltas. The western European population studied here is one of the isolated populations in Europe and includes the entire area of the Netherlands, parts of Flanders (Belgium), and East Frisia (Germany).

The hunting technique of the Pond Bat ranges from water trawling to aerial hunting. Earlier morphological studies suggested that pond bats are specialists on nonbiting midges (Diptera: Chironomidae; Britton et al. 1997; Sommer and Sommer 1997). Adult midges emerging from pupae are caught in flight while emerging from the water surface. Later morphological studies (Ciechanowski and Zapart 2012; Krüger et al. 2012), however, showed that pond bats, like many European bats (Vaughan 1997; Safi and Kerth 2004), have much more diverse diets that can include up to 10 different arthropod orders. Each prey species has its own characteristics including size, thickness of exoskeleton, flight ability, speed, and habitat.

### Study period and sites.

**Fig. 1.**
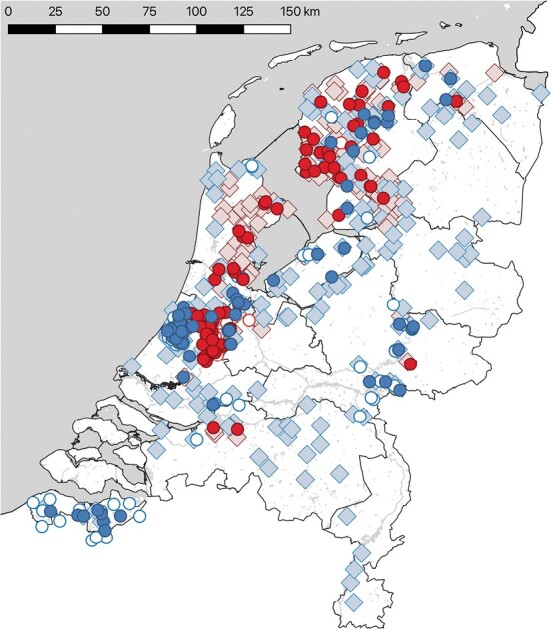
The summer distribution of the Pond Bat (*Myotis dasycneme*) population in the Netherlands (52°7ʹ57.5″N, 5°17ʹ28.6″E). The 114 locations where pond bats were captured (during the period 2003–2017) above waterways are indicated with filled circles. On the locations indicated with red (dark grey) circles, predominantly (>60%) females were caught; on the locations indicated with blue (grey) circles, predominantly (>60%) males were caught. The 106 open circles represent locations where mist nets were operated but no pond bats were caught. These locations are interpreted as being predominantly male or female based on nearby roost characteristics, but not used in analyses. Roosts used during the May–September months are indicated by light blue (male) and pink (female) diamonds, based on historic data (since 1900) and personal observations by the first author.

Data for this research were mainly collected between 2003 and 2012, with some extra samples collected up through 2017. A wide range of water-bound habitats throughout the Netherlands was sampled including marshes, lakes, rivers, wetlands, and waterways (Fig. 1). During 471 capture nights, mist nets were always used in combination with the tubing method for capturing water-trawling bats above the water surface without hoisting the mist net (Haarsma and van Alphen 2009a). Authorization of bat capture was provided by the Netherlands Food and Consumer Product Safety Authority (NVWA) over the entire sampling period (‘ontheffing Flora en Faunawet FF/75A/2016/048' assigned to the Dutch Mammal Society). For each site, the ratio of adult males and females was determined based on capture results. On 134 nights no adult pond bats were captured. Young born in the year of capture were excluded from this calculation, as they often live in the same habitat as their mothers. A total of 114 locations along waterways resulted in a successful capture effort, and for these waterways, water depth and soil type were determined. Water depth and soil type were based on the top 10 vector maps (www.pdok.nl/geo-services) with information about both variables. Peat was the most common soil type. All soil types were transformed into a numerical value by expressing soil into two binary categories: peat or other.

### Pellet collection and scoring of reproductive status.

Each captured bat was placed in a separate cotton holding bag until it was weighted, sexed, and the reproductive status and age were assessed by observation of external characteristics such as the coloration, size of the nipples, size of the abdomen, size and filling of the testes (Haarsma 2008; Haarsma and van Alphen 2009b). Bats were released following data collection. For females, sexually immature, sexually mature, pregnant, lactating, and post-lactating states were defined. These were then divided into two groups based on reproductive investment: (1) no investment = immature, sexually mature, or post-lactating; (2) investment = pregnant or lactating. For males, immature, mature, and sexually active states were used. Similar groups were made based on reproductive investment: (1) no investment = immature or sexually mature; (2) investment = sexually active.

Fecal pellets were collected from each separate holding bag and stored at room temperature until further analysis. Although one sample from one animal may sometimes consist of multiple (smaller) pellets rather than a single pellet, we prefer to use the term “pellet” (singular) instead of feces sample. We selected a total of 857 complete fecal pellets that were randomly divided into two subsets: 604 pellets for microscopic analyses and 253 pellets for DNA extraction (see below). The capture dates of all individuals were categorized into four periods (termed “season”), with each period encompassing approximately 25% of all data points (I = end of hibernation till May 20; II = May 21 to June 29; III = June 30 to July 30; and IV = July 31 until hibernation).

### Light microscopic analyses of fecal pellets.

A total of 604 pellets were analyzed from 25 males and 44 females, of which 69 consisted mainly of “hair balls.” A hair ball consisted predominantly of hairs, resulting from grooming in the roost. Such hair balls also consisted of several mites (e.g., *Spinturnix* spp.) and other skin parasites. These hair balls were excluded from further analyses, retaining 365 pellets from females and 170 from males (535 in total). Before dissecting, the dry weight of each fecal pellet was measured with an electronic scale. Details of all pellets analyzed are provided in a data archive (Haarsma et al. 2022). All pellets were soaked for 48 h in a mixture of 70% ethanol and glycerin. All samples were then dissected under a Carl Zeiss Discovery V20 stereomicroscope (30× to 80×). Characteristic insect fragments were mounted on microscopic slides in Euparal for further examination. Identifications were made by matching fragments to illustrations in arthropod identification keys (Shiel et al. 1997; Krüger et al. 2020). The relative abundance of prey groups was estimated by counting organs of which an individual insect has only a limited number, such as an antennal base (Chironomidae, Lepidoptera, Trichoptera), entire antenna (either aristate, lamellate, clavate, or stylate), eye rings (Trichoptera and Lepidoptera), halters (Diptera), tarsal claws, and tips of proboscis (all larger prey groups), and dividing these by the total number of these fragments per insect. The sex and developmental stage of the prey was determined whenever possible based on organs such as parameres, cornuti, eggs, crochets of prolegs, and exuvia (see Supplementary Data SD1 for examples). Prey items can remain in the digestive tract for a long time (Rabinowitz and Tuttle 1982; Staliński 1994). We therefore ignored nondiscriminating fragments, such as moth scales and pieces of exoskeleton, of which we assumed had been in the digestive tract for more than 24 h (Robinson and Stebbings 1993).

The known diet of pond bats includes Chironomidae as the dominant prey group, with a wide variety of additional prey. We grouped all recorded prey into nine taxonomic groups: five insect orders (Coleoptera, Hemiptera without infraorders Cicadomorpha and Aphidomorpha, Lepidoptera, Neuroptera, and Trichoptera); three mutually exclusive sub- and infraorders of Diptera (Brachycera, Culicomorpha, and Tipulomorpha), and a group termed “others” that contains the rarely found orders Araneae, Megaloptera, Hymenoptera, and Ephemeroptera—and the infraorders of Cicadomorpha and Aphidomorpha (Hemiptera). We then converted the diet into four quantitative expressions or indices of species diversity. The first two were estimated using the Shannon index (*H*) and Pielou’s evenness (equitability, *E*) following Magurran (2004). Structural diversity was described by the Shannon index, where *p*_*i*_ is the proportion of individuals in each of our nine prey groups:


$$\begin{array}{l} H=-\sum p_{i}\ln p_{i} \end{array}$$ (1)

*H* is a proportional index of diversity on a scale between 0 and ∞, where low values signal low diversity. In addition, evenness was calculated, where *S* is the number of prey groups in each sample:


$$\begin{array}{l} E=\frac{H}{\ln(S)} \end{array}$$ (2)

The evenness index is on a scale of 0–1, where 1 is perfectly even, which would be achieved if all prey groups were equally abundant. Third, the proportion of pupae (*P*_P_) of chironomids was calculated by dividing number of pupae by the total number of organisms of all species (“*N*”) in each sample. Lastly, we calculated the average length, width, and dry weight of each prey group by measuring specimens in our reference collection of prey species following the protocol of Gilbert (2011). Estimated weights per prey taxon and developmental stage are given in Haarsma et al. (2022). Based on our estimation of the relative abundance of each prey group, we calculated the average prey weight (APW in mg) for each pellet.

### DNA extraction from fecal pellets.

We analyzed a total of 253 fecal pellets with DNA metabarcoding. A total of 93 pellets (31 males and 62 females) did not yield results and were excluded from further analysis. The remaining pellets were derived from 95 females and 65 males. Details of all pellets analyzed are archived in Haarsma et al. (2022). The pellets were ground to a fine powder in liquid nitrogen with a mortar and pestle using the protocol of the Qiagen QIAamp DNA Stool Mini Kit in a special Ancient DNA facility dedicated to work with samples from degraded DNA and following established protocols to avoid contamination such as the inclusion of extraction blanks (Cooper and Poinar 2000). Subsequently, aliquots of each extraction were further purified using Promega PCR purification columns.

### PCR amplification of COI and 16S mini-barcodes and library preparation.

Amplifications of the ~313-bp-long mitochondrial *COI* mini-barcoding marker were performed using forward primer ZBJ-ArtF1c 5ʹ-AGATATTGGAACWTTATATTTTATTTTTGG-3ʹ and reverse primer ZBJ-ArtR2c 5ʹ-WACTAATCAATTWCCAAATCCTCC-3ʹ (Zeale et al. 2011). The ~157-bp-long mitochondrial *16S* barcoding marker was amplified using the forward primer P7_FO-16S 5ʹ-RGACGAGAAGACCCTATARA-3ʹ and P7_R0-16S 5ʹ-ACGCTGTTATCCCTAARGTA-3ʹ (Elbrecht et al. 2016). Primers were labeled for DNA metabarcoding with IonExpress labels. The PCR was carried out in 30 µl reactions containing 0.20 µl Qiagen Taq 5 u/µl, 3 µl 10× Qiagen buffer, 2 µl 2.5 mM dNTPs, 0.5 µl 10 µM forward primer, 0.5 µl 10 µM reverse primer, 1.5 µl 25 mM MgCl_2,_ 0.5 µl 10 mg/ml BSA, 19.80 µl MiliQ, and 2 µl template. Amplifications were performed using the following PCR program: 5-min denaturation at 95°C followed by 40 cycles of 20-s denaturation at 95°C, 20-s annealing at 50°C, and 1-min elongation at 72°C. Final elongation was conducted at 72°C for 7 min on a C1000 Biorad PCR machine.

### Ion Torrent sequencing.

Primer dimer and other contaminants were removed by using 0.9× Ampure XP beads (Agencourt) to which the PCR products were bound. The beads were washed with 150 µl 70% EtOH twice and resuspended in 20 µl TE buffer. Cleaned PCR products were quantified using an Agilent 2100 Bioanalyzer DNA High-sensitivity chip. An equimolar pool was prepared of the amplicon libraries at the highest possible concentration. This equimolar pool was diluted according to the calculated template dilution factor to target 10–30% of all positive Ion Sphere Particles. Template preparation and enrichment were carried out with the Ion One Touch 200 Template kit with use of the Ion One Touch System, according to the manufacturer’s protocol. Quality control of the Ion One Touch 200 Ion Sphere Particles was done with the Ion Sphere Quality Control kit using a Life Qubit 2.0. The enriched Ion Spheres were prepared for sequencing on a Personal Genome Machine (PGM) with the Ion PGM 200 Sequencing kit as described in the protocol and deposited on an Ion-314 chip (520 cycles per run) in three consecutive sequencing runs.

### Sequence analysis and data filtering.

Reads obtained from Ion Torrent sequencing were automatically sorted into separate sequence files based on the MID labels by the Ion Torrent software. The reads were further processed with PRINSEQ (version 0.20.3; Schmieder and Edwards 2011) with the following settings: a minimum read length of 100 bp, trimming to 140 bp, minimum mean quality of Q24 per read, additional trimming of 3ʹ end bases with a *Q* lower than 24, and removal of full duplicate sequences. Filtered reads were clustered into Operational Taxonomic Units (OTUs) defined by a sequence similarity of at least 97% using CD-HIT-EST (Li and Godzik 2006)—singletons were omitted. For each cluster the representative sequences were BLASTed with the NCBI-blast+ software package (version 2.2.28+; Camacho et al. 2009) against either the NCBI GenBank nucleotide database or a custom database containing all Arthropod sequences located on the Barcode of Life Database (Ratnasingham and Hebert 2007). BLAST hits were filtered according to the following criteria: minimum hit length of a 100 bp; minimum hit similarity of 97%; and a maximum *e*-value of 0.05. Reference databases of Dutch species such as http://www.nederlandsesoorten.nl/ were used to check whether a species had been recorded for the Netherlands. All species not yet known for the Netherlands were reduced to genus level or to the family level if the genus is also unknown to occur.

### Weather data.

We downloaded weather data from the website of the Royal Netherlands Meteorological Institute (KNMI, https://www.knmi.nl/nederland-nu/klimatologie/uurgegevens). The data set consisted of hourly data of temperature, wind speed, and precipitation. Only the first 2 h after sunset were used for analyses. Air temperature is known to affect the flight activity of insect prey, and thus has a large influence on bat activity (Rydell 1989; Russ et al. 2003). Wojciechowski et al. (2007) considered a temperature of 8°C as the average minimum threshold value for foraging activity of European bats.

### Statistical analyses.

The probability that a captured adult Pond Bat was female was analyzed with a binomial regression analysis with water depth and soil type (peat or other) as explanatory variables, and year and site as random effects, using the *glmer* function in R. Variation among pellets in prey group evenness, Shannon index, proportion of prey that were Chironomidae pupae, and average prey size was analyzed first with regression analyses with sex and season as variables. Post hoc Tukey tests were applied to test for pairwise differences between all sex–season combinations. We also performed separate analyses per sex with data only from the periods in which females were pregnant (periods I and II) and males were observed to be sexually active (periods I, III, and IV). These sex-specific analyses tested effects of reproductive status, water depth, soil type, temperature, wind speed, and pellet weight. Continuous variables were normalized (mean = 0, *SD* =1) prior to analyses. Year was included as a random factor in all cases. Evenness and the Shannon index were analyzed with *lme* and an exponential variance function to deal with heteroscedasticity. The probability that a prey item was a Chironomidae pupa was analyzed with a binomial *glmer*. Average prey size per pellet was ln-transformed prior to *lme* analysis. Whether the presence of insect families in the pellet samples analyzed with DNA metabarcoding differed between the four sex–maturity combinations was explored with bivariate cluster analysis (Maechler et al. 2022).

## Results

Based on capture observations of adult males and females on 114 water-bound sites (Fig. 1)—spread over eight different soil types, various water depths, all seasons, and over a period of 15 years—we calculated the chance that a caught Pond Bat was female. We found that this chance was significantly higher in peatlands than in other habitats. Females might also prefer shallow water, but this effect is not statistically significant (Supplementary Data SD2).

### Diet composition of male and female pond bats

#### Morphological analyses.

In total, 13 different taxonomic groups were identified in the fecal samples analyzed. Small nonbiting midges (Diptera: Culicomorpha—for females 95.6% and males 94.7% of all samples) and moths (Lepidoptera—females 40.5% and males 49.4% of all samples) were found to be the most frequently observed prey groups. In addition, intermediate-sized flies (Diptera: Brachycera), craneflies (Diptera: Tipulomorpha), and caddisflies (Trichoptera) also occurred in many pellets (20–30%). Based on the presence of nearly complete wing, leg, and antenna fragments in the fecal pellets, most prey belonging to the suborder Brachycera could be identified as dung flies (Diptera: Scathophagidae). Other Diptera families identified included biting midges (Diptera: Ceratopogonidae), mosquitos (Diptera: Culicidae), house flies (Diptera: Muscidae), hoverflies (Diptera: Syrphidae), and dagger flies (Diptera: Empedidae). Similar morphology-based identifications could be made for dung beetles (Coleoptera: Scarabaeoidea) and water beetles (Coleoptera: Dytiscidae and Hydrophilidae). In total, 23 different insect families could be identified based on morphology. The size of consumed prey varied from 0.6 to 30 mm, with a weight of 0.6–35 mg. We found 19 larvae of Lepidoptera in the pellets analyzed—probably gleaned from land—and 167 pupae of Chironomidae—possibly captured with a water-trawling technique. In 30 (27 females, three males) samples these pupae could be identified up to genus level, and based on the racket-like epaulets along their abdomen, they belonged to *Glyptotendipes* spp. The sex of the prey, males with typical genital structures and females with eggs, could be identified in 171 cases; Coleoptera (female:male = 5:0), Diptera: Tipulidae (60:0), Hemiptera: Corixidae (25:0), Coleoptera: Dytiscidae (2:4), Trichoptera: Hydropsychidae (3:0), Lepidoptera (30:27), Coleoptera: Scarabaeidae (2:6), Diptera: Scathophagidae (2:0), Diptera: Tabanidae (3:0), Neuroptera (3:0), and Hymenoptera (1:0).

#### Molecular analyses.

DNA metabarcoding confirmed the presence of several families such as dung flies and dung beetles in the fecal samples and added resolution up to family and sometimes even species level, especially for moths (Lepidoptera: Crambidae, Geometridae, Noctuidae) and caddisflies (Trichoptera: Hydropsychidae, Leptoceridae, Lymnephildae, Phryganeidae; data archived in Haarsma et al. 2022). The genus *Glyptotendipes* was found with DNA metabarcoding (f:m = 22:18), but the technique used cannot distinguish between life stages (pupae or adult) of the prey. The water beetles (Coleoptera: Dytiscidae and Hydrophilidae) could be identified to genus level, and according to GenBank all belonged to the genus *Rhantus*. Extra resolution could be added to genus level within other infraorders such as Tipulomorpha (Diptera), Gerromorpha (Hemiptera), and Muscomorpha (Diptera). In total, we identified 10 different insect orders, 52 families, and 161 species with metabarcoding.

#### Comparison between morphological and molecular analyses.

**Fig. 2.**
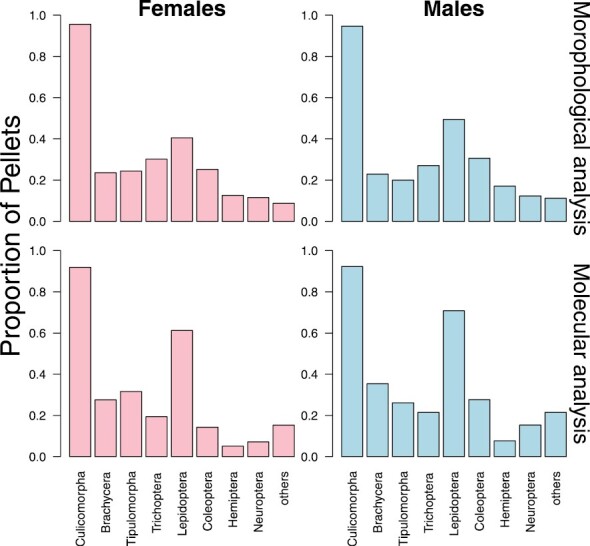
Proportion of pellets with at least one prey item detected for female (left) and male animals (right), separately per taxon. Top row: based on microscopy. Bottom row: based on DNA metabarcoding. Underlying data can be found in Haarsma et al. (2022).

The results of metabarcoding and microscopy, summarized at the level of the nine main taxonomic groups, yielded similar results (Fig. 2; raw data in Haarsma et al. 2022). Nonbiting midges (Diptera: Culicomorpha) were found in 38.7% (females) and 29.8% (males) of the samples analyzed with DNA metabarcoding, compared to 50.2% (females) and 47.8% (males) with morphological analyses. Moths (females: 25.5% and males: 34.7%) were identified in more samples with molecular analyses than with morphological analyses, while Trichoptera and Hemiptera were consistently found in fewer samples.

#### Seasonal differences in diet composition of pond bats.

**Fig. 3.**
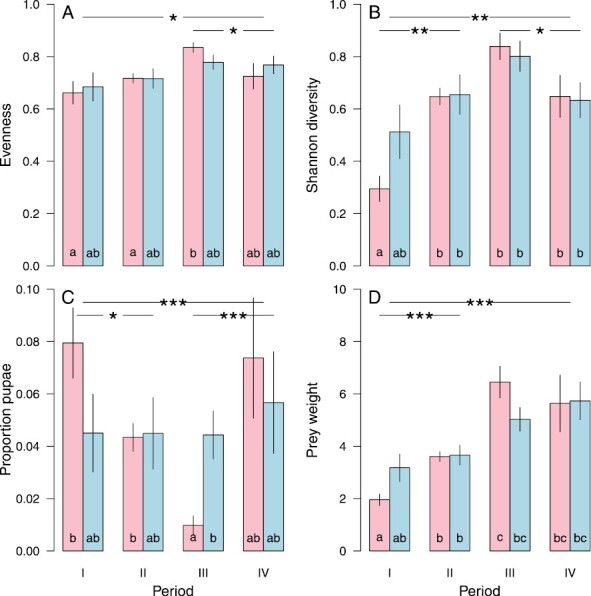
Evenness (A) in prey over the nine taxonomic groups, Shannon diversity index (B), the proportion of all prey that are Chironomidae pupae (C), and average prey weight in mg per pellet, and separate per Pond Bat sex (left bars refer to females, right bars to males) and time period (I = end of hibernation till May 20, II = May 21 to June 29, III = June 30 to July 30, IV = July 31 until hibernation). Bars indicate standard errors. Different letters (a, b, c) indicate significant differences between bars. Significant differences between the first two and the latter two periods (and between periods I and II, and between III and IV) are indicated on top (see details in Supplementary Data SD3).

Based on morphological analysis, APW, pupae proportion (*P*_p_), and diet diversity indices *H* and *E* (based on the taxonomic groups as archived in Haarsma et al. 2022) showed temporal variation among the four seasonal periods (Fig. 3). This variation was larger between the four periods than between the sexes (Supplementary Data SD3), except for prey weight. Both evenness and Shannon diversity index were small in periods I and II and larger in period III. Taxonomic groups differ in size, with Culicomorpha being the smallest-sized prey. The pellets were more or less similar-sized, resulting in the fact that a pellet with a high number of small prey items (e.g., Culicomorpha) tended to have a low evenness in combination with a low Shannon index. In period I females tended to eat smaller prey items than males, while in period III males tended to show a lower evenness and Shannon index than females (Fig. 3). The largest seasonal changes were observed in the APW, which increased drastically from small prey with an average weight of 0.001 g in spring to larger prey with an average weight of 0.008 g to a maximum of 0.025 g later in the year (*R*^2^ = 0.067, *P* = 0.000). The relative frequency of heavier prey species of land and water beetles (Coleoptera: Carabidae, Dytiscidae) and moths (Lepidoptera: Crambidae, Geometridae, Noctuidae) in the fecal pellets of both male and female pond bats increased toward the end of the summer, which is the start of the mating season. However, these seasonal effects were such that they cannot explain the observed variance in the diet of males and females—they merely indicate that both sexes change their diet during the year. We therefore examined differences between the diet of males and females within each season separately.

#### Differences in diet composition linked to reproductive investment.

**Fig. 4.**
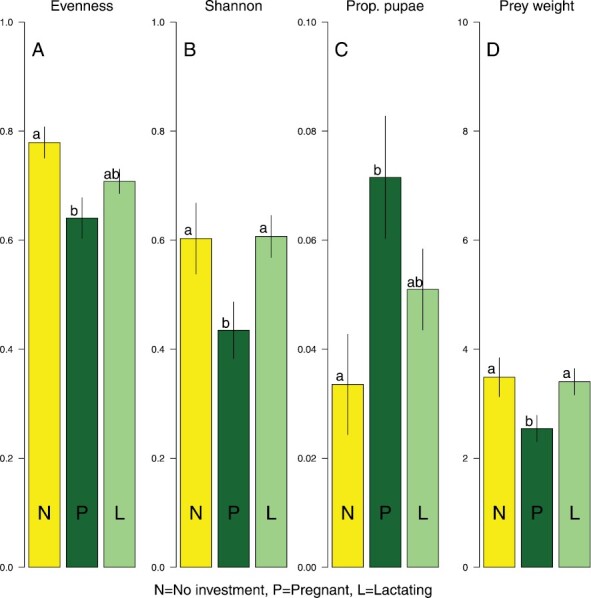
Pellet characteristics of female pond bats in periods I and II. Evenness (A) in prey over the nine taxonomic groups, Shannon diversity index (B), the proportion of all prey that are Chironomidae pupae (C), and average prey weight in mg (D) per pellet—and separated for pregnant females, lactating females, and females that were not currently investing in reproduction (i.e., post-lactating as well as immature females). Bars indicate standard errors. Different letters (a vs. b) indicate significant differences between bars. For analyses including other explanatory variables, see Supplementary Data SD4.

To account for the effect of temporal variation in the diet of females and focus on the effect of “reproductive investment,” only pellets collected in periods I and II were included in the analyses (because pregnant females were only found in those two periods). The effect of female reproduction investment (investment = pregnant and lactating; vs. no investment = immature, mature, and post-lactating) is significant for all diet diversity indices and APW (based on morphological data; Supplementary Data SD4). Prevailing temperature had a minor, but significant, effect on variation in observed prey in the diet. With higher temperatures the diversity (*H*), evenness (*E*), and prey weight tended to increase, while the number of pupae tended to decrease. The most pronounced overall effect was found with the proportion of pupae (*P*_p_), indicating pupae are a larger part of the diet during pregnancy and to a lesser extent while lactating (Fig. 4C). Both the Shannon (*H*) and evenness (*E*) indices are significantly lower in pregnant bats than in immature and post-lactating females (Fig. 4A,B, Supplementary Data SD4), implying consumption of a large number of small prey items. This was also found with metabarcoding (Supplementary Data SD5), which showed that Chironomidae (Diptera) are a large part of the diet of females with reproductive investment: statistically significant higher proportion of pellets with Chironomidae compared to nonreproductive females (χ^2^ = 58.8, d.f. = 1, *P* < 0.001). Tipulidae (Diptera; χ^2^ = 6.2, d.f. = 1, *P* = 0.013) and Geometridae (Lepidoptera; χ^2^ = 5.9, d.f. = 1, *P* = 0.015) also occurred more often in pellets of reproductive females than in those of nonreproductive females. Cluster analysis based on the family-level presence–absence data per pellet showed large overlap between the four sex–reproduction combinations (Supplementary Data SD6).

Sexual activity of males occurred in periods I, II, and IV. However, we did not find a significant effect of reproduction (sexually active yes or no) on their diet (based on morphological data; Supplementary Data SD7). Water depth, soil type, wind speed, and sexual age (immature and mature) did not have an effect on the diet composition. However, temperature did have a small (but significant) positive effect on both the Shannon index (*H*) and the APW.

Metabarcoding results showed that Noctuidae (Lepidoptera), Chironomidae (Diptera), and Chrysopidae (Neuroptera) were a smaller part of the diet of sexually active males (Supplementary Data SD5) than of males without reproductive investment. For example. Corixidae (Hemiptera) and Scathophagidae (Diptera) were eaten more frequently by sexually active males. However, caution should be taken with the results of the males—we observed only a few hunting adult males during suboptimal weather conditions, such as strong wind (>3 bft or >5 m/s) and low temperatures (<12°C) during periods I and II. During such nights adult females were found to be active in their hunting habitat, while adult males were not observed, probably because males remained in torpor in their roost.

## Discussion

Combined morphological and molecular analysis of pellets collected from individual bats provided a complete description of the spatiotemporal diet differences in prey diversity and prey weight between male and female pond bats. Females are mainly found in peatlands with shallow water, while males not only live in similar habitat as the females, but also occur on other soils. Observed spatiotemporal differences in diet were caused not merely by differences in habitat, but in the case of female bats also by differences in season and reproductive status.

Optimization of food intake involves at least three attributes of the system: (1) local abundance of prey items; (2) variation in seasonal availability of prey items related to soil type and small temperature differences due to habitat structure; and (3) variation in energy requirements of the hunter according to sex and life stage—lactating females, for instance, need a higher energy intake than males. Although bats are often considered opportunists with the ability to capture a large diversity of prey (e.g., Clare et al. 2009), each bat species has specialized adaptations in wing design, echolocation, and feet morphology to help locate and grasp prey (see Haarsma and Siepel 2013 and citations therein)—the most energy-efficient hunting method is probably associated with the most effective use of these adaptations. However, temporal limitations also need to be considered—for instance, the relative mass of and embryo or embryos can weigh up to 30% of the body weight of an adult female (Hayssen and Kunz 1996), making pregnant bats more ungainly and less likely to capture large and agile prey such as moths.

A second hypothesis for explaining the differences in diet found between both sexes might be the handling time per prey unit. Bats are known to have different handling times for different prey groups (e.g., Aguirre et al. 2003; Koselj et al. 2011)—however, studies measuring such handling times are rare. We assume that prey with a hard exoskeleton take more handling time than prey with a soft exoskeleton, and pregnant bats might favor a shorter handling time due to their higher energy demands. Without numerical information about handling time, we were not able to correct our data for biases caused by handling time, but our results imply that this might be at play here, in combination with prey availability. Further experimental validation is needed to test this hypothesis.

Temporal variation in diet was larger between the four study periods than between the sexes. By examining differences within each season, we were able to highlight the differences between male and female diet. We found the largest dietary shift in period I and II, the period with the highest reproductive investment of the females. By using pellets from known individuals, we were able to demonstrate that the diet of pregnant females is different to that of females with no reproductive investment.

In several aspects, our data are consistent with earlier diet studies of the Pond Bat. We confirmed that nonbiting midges (Diptera: Chironomidae) are their bulk prey (Britton et al. 1997; Sommer and Sommer 1997; Ciechanowski and Zapart 2012; Krüger et al. 2012). The frequent occurrence of Chironomidae pupae indicates their use of water trawling. The other prey groups we could identify reflect the adaptability of *M. dasycneme* concerning its hunting techniques. Besides prey groups including Trichoptera and Lepidoptera (mainly hunted by aerial hunting), we also found several land-related prey, and even prey that must have been gleaned including caterpillars (Lepidoptera), dung flies (Scathophagidae), and dung beetles (Scarabaeidae), suggesting that pond bats forage not only above water but also above land. The occurrence of these prey taxa was confirmed with DNA metabarcoding, which also yielded insight in the actual species or genus of the prey.

Results obtained for aquatic foraging habitats identified arthropod communities (e.g., Diptera, Trichoptera, Lepidoptera) that were very similar with those found earlier across Europe (Krüger et al. 2012). This is not surprising as these habitats have similar landscape features within the entire Pond Bat distribution area. However, local variability in diet was also found (Almenar et al. 2008; Clare et al. 2011; Nissen et al. 2013; Andriollo et al. 2019). The observed variation in food items in this study is probably best explained by differences in the availability and abundances of food resources that, in turn, relate to climatic and biotic conditions. Regional differences in the intensity of agriculture correspond with different levels of nitrogen compounds emitted and correlate to eutrophication of water systems. This will influence the diet of local pond bat populations hunting in these areas.

### Observed contrast in diet between the Netherlands and other European countries.

Prey taxa detected in the diet of Dutch pond bats did not always correlate with findings of other studies. In Poland, small caddisflies (Trichoptera: Hydropsychidae, Leptoceridae, Lymnephilidae, Phryganeidae) were found as the bulk prey of pond bats studied between the end of June and the end of July (Ciechanowski and Zapart 2012). In contrast, we observed an increase in large prey such as dung flies (Diptera: Scathophagidae), water beetles (Coleoptera: Carabidae, Dytiscidae), and moths (Lepidoptera: Crambidae, Geometridae, Noctuidae) for these 2 months. The Polish study was carried out in a nearly pristine natural area, whereas the Dutch pond bats forage in a heavily cultivated landscape filled with intensively managed agricultural fields, which might explain the differences in diet found.

### Pregnant or lactating bats have different diet than nonreproductive females.

Both pregnant/lactating and nonreproductive bats show opportunistic feeding behavior, concentrating on the temporarily and locally most abundant prey species. Bats in each reproductive category have the same tendency for this behavior. However, we found other differences between the diet of pregnant and nonreproductive females. Instead of gathering heavier prey, pregnant (and lactating) females actually tended to eat larger numbers of nonbiting midges (Diptera: Chironomidae) than nonreproductive females. Morphological analyses showed that those midges included a large number of pupae, probably gathered from the water surface with the trawling hunting technique. Such prey types can be expected in large quantities in typical female habitat such as locations intersecting with peatland (in combination with shallow water bodies). This result is consistent with our assumption that brown shallow water warms faster, and thus produces a relatively large amount of prey emerging from the surface. Our findings support the hypothesis that pregnant pond bats are more ungainly and less likely to capture large and agile prey such as moths.

### Male and female pond bats have different diets.

We found significant differences between the diet of males and females, and these differences become more pronounced when reproductive investment is taken into account. Such diet differences are also known from other temperate zone bats (Acharya 1995; Whitaker 2004). Hunting female pond bats can be found in all weather conditions and their distribution in this study was found to be closely linked to shallow brownish water. In summer, male pond bats tend to remain in torpor during suboptimal weather conditions, as documented during emergence surveys of roosts in the context of the national Pond Bat monitoring program (following the protocol of Haarsma and Tuitert 2009; Haarsma A.-J., personal observations). This behavior has also been observed in other bat species (Grinevitch et al. 1995; Dietz and Kalko 2006). The gestation and lactation periods of female pond bats in temperate regions are very likely triggered by the availability of food resources (Arlettaz et al. 2001; Montiel et al. 2011). Correct ontogenetic timing is very important for the survival of the young as they cannot survive long without milk. A habitat with a high diversity and abundance of small bulk prey species, in addition to the presence of early emerging bulk prey such as midges (Diptera: Chironomidae), was found to be important for female pond bats in this study. The availability of emergent aquatic insects was also found to be important for females of other bat species (Fukui et al. 2006). As aquatic insects need shelter from wind during their emerging phase, a sufficient number of sheltered waterways for hunting are important during suboptimal weather conditions.

### Detailed information on the sexual status of the producer of each pellet.

Multiple studies report on linking food competition and habitat use to interspecies competition among bats (Arlettaz 1999; Arlettaz et al. 2001; Lee and McCracken 2004). In addition, observations of intraspecific competition have been published, especially for females during periods with high reproductive investment (Mackie and Racey 2007; Preatoni et al. 2011; Lintott et al. 2014). Studies showing a dietary change during reproductive investment are rare. Because of overlapping timing of reproductive stages, as shown in Haarsma and van Alphen (2009c), individuals from the same roost can be both pregnant and lactating. Unlike other studies based on pellets collected from unknown individuals in a nursery roost (Goiti et al. 2004; Ciechanowski and Zapart 2012), we collected pellets from individual bats and therefore were able to link diet and reproductive status more accurately.

### Conservation implications.

The Pond Bat is rare and occurs at low density across most of its European range. In their analysis of population trends based on emergence counts of 70% of the Dutch population, Haarsma and Koopmans (2018) have shown that both population size and colony size have declined over 39% since 2009. Similar trends are reported for other countries (Boye et al. 2004; Pētersons and Vintulis 2020). Better understanding of the diet of this particular species obtained here can help to improve its conservation. Two new insights obtained in this study can be particularly useful. First, the diet of the Pond Bat in the Netherlands as reconstructed in this study was surprisingly diverse, comprising both aquatic and terrestrial insect taxa. Aquatic species such as midges (Diptera: Chironomidae) are not doing well in the Netherlands, likely due to reduced nutrient levels in surface waters (Hallmann and Jongejans 2021) and high levels of neonicotinoid pesticides in combination with other pesticides in the surface water (van Dijk et al. 2013; Vijver and van den Brink 2014). Hallmann et al. (2014) found a correlation between neonicotinoid pesticide use and the decline of insectivorous birds. A ban on the use of neonicotinoids, as issued for imidacloprid in 2015, is expected to help to restore aquatic insect diversity. To help in providing access to emergent pupae of midges, water management organizations should also aim to keep canals clear of water plants during the breeding season as hunting pond bats avoid water surfaces entirely covered by duckweed (Boonman et al. 1998) or other organic matter (Mackey and Barclay 1989).

Land insect species newly discovered in the diet of pond bats included dung flies (Scathophagidae) and dung beetles (Scarabaeidae) associated with feces of livestock such as cows and horses. In other countries in Europe, pond bats have a much less diverse diet that does not include as many insect species associated with intensive agriculture. It seems that pond bats incorporate this alternative prey because of their relative abundance, whereas agricultural intensification reduces their preferred aquatic prey at the same time.

## Supplementary Data

Supplementary data are available at *Journal of Mammalogy* online.

**Supplementary Data SD1.**—The sex and developmental stage of prey were estimated based on fragments of organs such as parameres, crochets (prolegs), and genitalia. Photographs by A-JH.

**Supplementary Data SD2.**—Binomial regression analysis (*glmer* with site and year as random factor) of the sex of caught pond bats. The factors peatland and normalized water depth were fixed effects.

**Supplementary Data SD3.**—Effect sizes (and their standard errors) of time period, sex, and their interactions. Females and periods I and II are incorporated in the intercept. Fitted regression models have year as a factorial random effect. In the regression analyses of evenness and the Shannon diversity index we dealt with heteroscedasticity by including an exponential variance function. The proportion of prey that were Chironomidae pupae was analyzed with a logit link function. Mean prey weight per pellet was ln-transformed prior to the analysis. Effect sizes that are significantly different from 0 are indicated in bold. See Fig. 3 in the main text for mean values and standard errors.

**Supplementary Data SD4.**—Effect sizes (and their standard errors) in regression analyses of prey diversity evenness, Shannon diversity index, proportion Chironomidae pupae, and ln-transformed mean weight of prey in pellets of female pond bats. Only pellets collected in periods I and II were included in the analyses, because pregnant bats were only caught in those two periods. In the analyses we test whether females that invest in reproduction (i.e., those that are pregnant or lactating) have different values than post-lactating or immature females. We also test whether mature (but not pregnant or lactating) females differ from immatures, and whether pregnant versus lactating females have different values. The continuous explanatory variables water depth, wind speed, temperature, and pellet weight were normalized (mean = 0, *SD* = 1) prior to analyses. Fitted regression models have year as a factorial random effect. In the regression analyses of evenness and the Shannon diversity index we dealt with heteroscedasticity by including an exponential variance function. The proportion of prey that were Chironomidae pupae was analyzed with a logit link function. Effect sizes that are significantly different from 0 are indicated in bold.

**Supplementary Data SD5.**—Percentage of pellets with at least one prey of a certain insect family (dotted lines demarcate insect orders), based on DNA metabarcoding analysis. Pellets of females are separated depending on whether a female was pregnant or lactating versus not showing such signs of reproductive investment (being either sexually immature, sexually mature, or post-lactating). Pellets of males are separated based on whether they showed signs of sexual activity versus not showing such signs (sexually mature and sexually immature).

**Supplementary Data SD6.**—Bivariate cluster plot based on insect family presence data resulting from DNA metabarcoding 160 pellets of the Pond Bat *Myotis dasycneme*. “fa” = female adult; “mj” = male juvenile; “ma” = male adults; “fj” = female juvenile (green).

**Supplementary Data SD7.**—Effect sizes (and their standard errors) in regression analyses of the prey diversity evenness, Shannon diversity index, proportion Chironomidae pupae, and ln-transformed mean weight of prey in pellets of male pond bats. Only pellets collected in periods I, III, and IV were included in the analyses, because sexually active males were only caught in those periods. In the analyses we test whether males that were sexually active have different values than those that were not. We also test whether mature (but not sexually) males differ from immatures. The continuous explanatory variables water depth, wind speed, temperature, and pellet weight were normalized (mean = 0, *SD* = 1) prior to analysis. Fitted regression models have year as a factorial random effect. In the regression analyses of evenness and the Shannon diversity index we dealt with heteroscedasticity by including an exponential variance function. The proportion of prey that were Chironomidae pupae was analyzed with a logit link function. Effect sizes that are significantly different from 0 are indicated in bold.

gyad096_suppl_Supplementary_Data_SD1

gyad096_suppl_Supplementary_Data_SD2

gyad096_suppl_Supplementary_Data_SD3

gyad096_suppl_Supplementary_Data_SD4

gyad096_suppl_Supplementary_Data_SD5

gyad096_suppl_Supplementary_Data_SD6

gyad096_suppl_Supplementary_Data_SD7

## Data Availability

Next-generation sequencing data are available at NCBI GenBank (http://www.ncbi.nlm.nih.gov/bioproject/680901). All other data sets are archived in Haarsma et al. (2022).
