# Supplementary figures and images for: Female pond bats hunt in other areas than males and consume lighter prey when pregnant
Source: J Mammal. 2023 Oct 16;104(6):1191–204. doi: 10.1093/jmammal/gyad096 (PMC10697422; doi:10.1093/jmammal/gyad096)

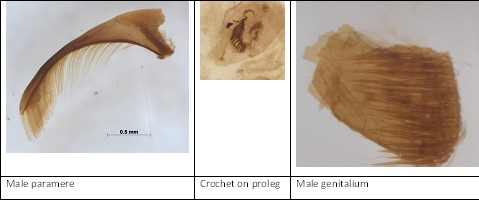

Supplement: gyad096_suppl_Supplementary_Data_SD1 [file gyad096_suppl_supplementary_data_sd1.jpeg]

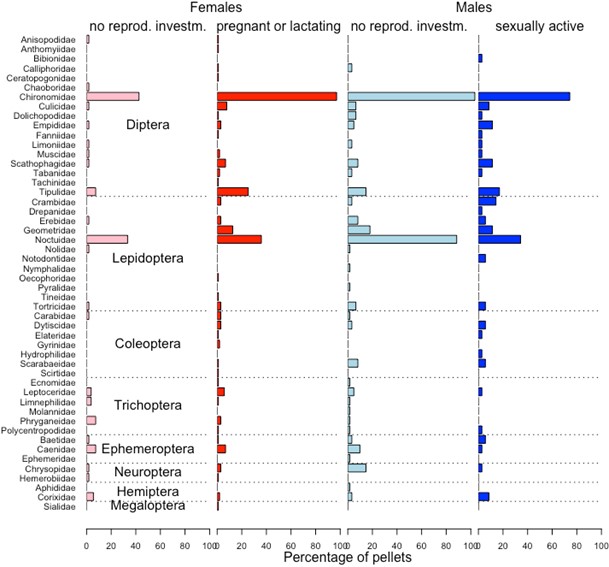

Supplement: gyad096_suppl_Supplementary_Data_SD5 [file gyad096_suppl_supplementary_data_sd5.jpeg]

# Cluster plot of insect family presence per pellet based on metabarcoding

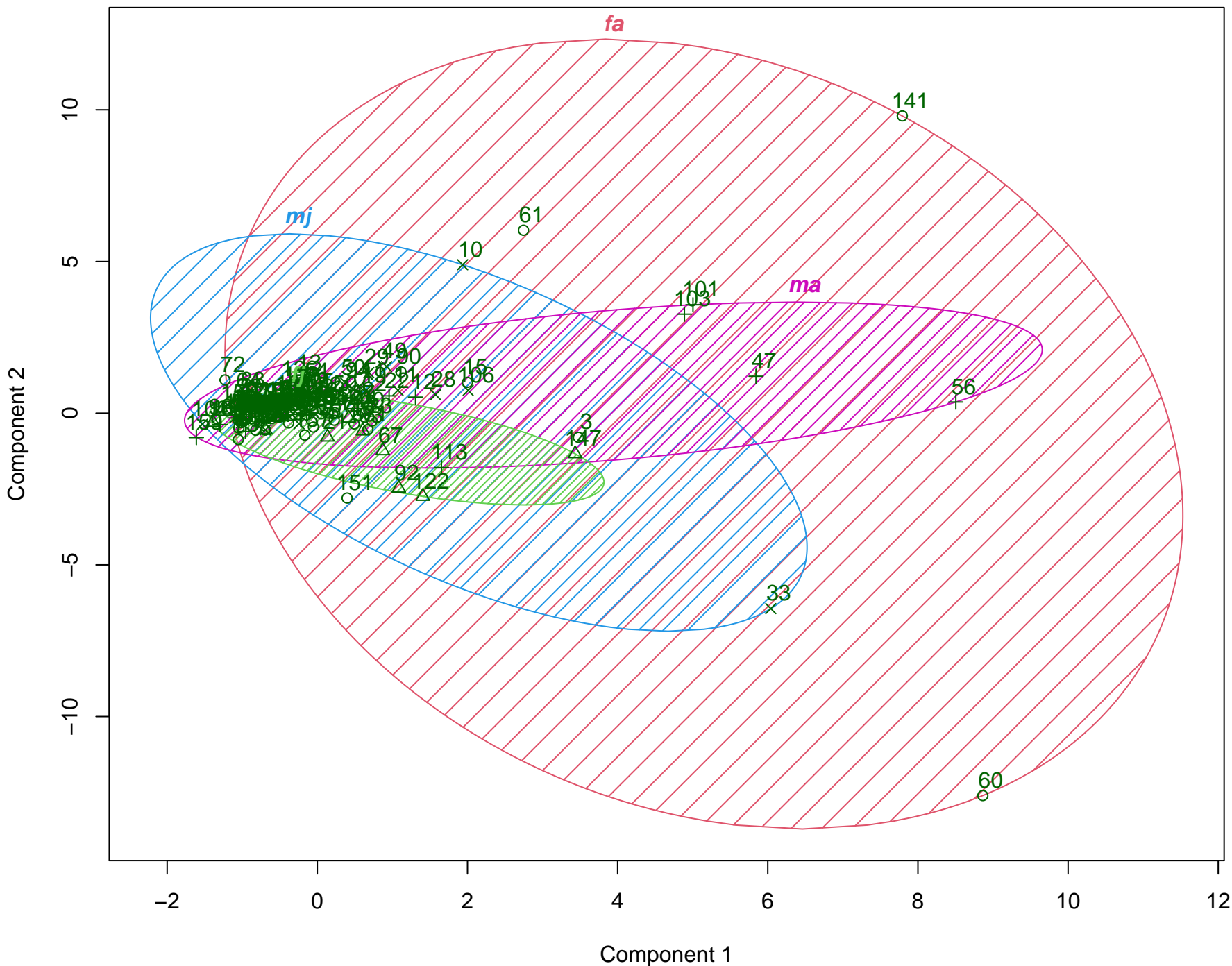

Supplement: gyad096_suppl_Supplementary_Data_SD6 [file gyad096_suppl_supplementary_data_sd6.pdf]
