# Supplementary material for: Female pond bats hunt in other areas than males and consume lighter prey when pregnant
Source: J Mammal. 2023 Oct 16;104(6):1191–204. doi: 10.1093/jmammal/gyad096 (PMC10697422; doi:10.1093/jmammal/gyad096)
Supplement: gyad096_suppl_Supplementary_Data_SD2 [file gyad096_suppl_supplementary_data_sd2.pdf]

```
Random effects:
 Groups Name        Variance Std.Dev.
 site   (Intercept) 7.8683   2.8050
 year   (Intercept) 0.5696   0.7547
Number of obs: 217, groups:  site, 114; year, 14

Fixed effects:
            Estimate Std. Error z value Pr(>|z|)
(Intercept)   0.1303     0.5943   0.219    0.826
peat          3.3655     0.8028   4.192 2.76e-05 ***
sdepth       -0.4296     0.3587  -1.198    0.231
```

Binomial regression analysis (*glmer* with year and site as random factors) of the probability that a caught pond bat was female.
Depth is the normalisation of water depth (mean 1.6065 m, standard deviation 0.5870 m).
