# Supplementary material for: Female pond bats hunt in other areas than males and consume lighter prey when pregnant
Source: J Mammal. 2023 Oct 16;104(6):1191–204. doi: 10.1093/jmammal/gyad096 (PMC10697422; doi:10.1093/jmammal/gyad096)
Supplement: gyad096_suppl_Supplementary_Data_SD3 [file gyad096_suppl_supplementary_data_sd3.pdf]

Effect sizes (and their standard errors) of time period, sex and their interactions. Females and periods I and II are incorporated in the intercept. Fitted regression models have year as a factorial random effect. In the regression analyses of evenness and the Shannon diversity index we dealt with heteroscedasticity by including an exponential variance function. The proportion of prey that were Chironomidae pupae was analysed with a logit link function. Mean prey weight per pellet was ln-transformed prior to the analysis. Effect sizes that are significantly different from 0 are indicated in bold. See figure 3 in the main text for mean values and standard errors.

Effect	Evenness	Shannon index	Prop. pupae	ln(Prey weight)
(Intercept)	**0.6892 (0.0246)**	**0.6520 (0.0392)**	**-2.5239 (0.1491)**	**0.6886 (0.0694)**
Male	0.0109 (0.0450)	0.0637 (0.0659)	-0.1801 (0.2145)	**0.2578 (0.1235)**
Periods 3&4	**0.0904 (0.0352)**	**0.1833 (0.0563)**	**-0.7382 (0.2082)**	**0.7069 (0.1067)**
Period 2 vs 1	0.0277 (0.0246)	**0.0931 (0.0335)**	**-0.2075 (0.0937)**	**0.2883 (0.0581)**
Period 4 vs 3	**-0.0548 (0.0252)**	**-0.0941 (0.0454)**	**0.8392 (0.1905)**	-0.0564 (0.0903)
Male:Periods3&4	-0.0176 (0.0559)	-0.0439 (0.0902)	0.6151 (0.3182)	**-0.3599 (0.1710)**
Male:Period2vs1	-0.0118 (0.0450)	-0.0614 (0.0658)	0.3896 (0.2149)	-0.1964 (0.1238)
Male:Period4vs3	0.0498 (0.0332)	0.0377 (0.0608)	**-0.9592 (0.2337)**	0.0853 (0.1178)
