# Supplementary material for: Female pond bats hunt in other areas than males and consume lighter prey when pregnant
Source: J Mammal. 2023 Oct 16;104(6):1191–204. doi: 10.1093/jmammal/gyad096 (PMC10697422; doi:10.1093/jmammal/gyad096)
Supplement: gyad096_suppl_Supplementary_Data_SD4 [file gyad096_suppl_supplementary_data_sd4.pdf]

Effect sizes (and their standard errors) in regression analyses of the prey diversity evenness, Shannon diversity index, proportion Chironomidae pupae and ln-transformed mean weight of prey in pellets of female pond bats. Only pellets collected in periods I and II were included in the analyses, because pregnant bats were only caught in those two periods. In the analyses we test whether females that invest in reproduction (i.e. those that are pregnant or lactating) have different values than post-lactating or immature females. We also test whether mature (but not pregnant or lactating) females differ from immatures, and whether pregnant females have different values than lactating ones. The continuous explanatory variables water depth, wind speed, temperature and pellet weight were normalized (mean=0, sd=1) prior to analyses. Fitted regression models have year as a factorial random effect. In the regression analyses of evenness and the Shannon diversity index we dealt with heteroscedasticity by including an exponential variance function. The proportion of prey that were Chironomidae pupae was analysed with a logit link function. Effect sizes that are significantly different from 0 are indicated in bold.

Effect	Evenness	Shannon index	Prop. pupae	ln(Prey weight)
(Intercept)	**0.8007 (0.0430)**	**0.6896 (0.0914)**	**-2.7892 (0.3179)**	**1.0985 (0.1532)**
Repro. Invest.	**-0.0873 (0.0342)**	**-0.1532 (0.0649)**	**0.4646 (0.2369)**	**-0.2334 (0.1111)**
Mature vs Imma.	-0.0379 (0.0302)	-0.0080 (0.0580)	-0.2323 (0.2185)	-0.0867 (0.0988)
Lactat. vs Pregn.	0.0133 (0.0198)	**0.0662 (0.0321)**	-0.0123 (0.0946)	0.0690 (0.0575)
Water Depth	-0.0167 (0.0177)	-0.0079 (0.0320)	-0.1372 (0.1162)	-0.0391 (0.0555)
Peat vs other soil	-0.0069 (0.0385)	-0.0096 (0.0772)	**-0.7589 (0.2534)**	-0.0408 (0.1305)
Wind speed	-0.0120 (0.0179)	-0.0183 (0.0345)	**0.2094 (0.1043)**	-0.0643 (0.0588)
Temperature	**0.0523 (0.0166)**	**0.1444 (0.0334)**	**-0.3506 (0.1290)**	**0.2555 (0.0559)**
Pellet weight	**-0.0878 (0.0172)**	**0.0547 (0.0260)**	0.0673 (0.0511)	0.0959 (0.0429)
