# Supplementary material for: Female pond bats hunt in other areas than males and consume lighter prey when pregnant
Source: J Mammal. 2023 Oct 16;104(6):1191–204. doi: 10.1093/jmammal/gyad096 (PMC10697422; doi:10.1093/jmammal/gyad096)
Supplement: gyad096_suppl_Supplementary_Data_SD7 [file gyad096_suppl_supplementary_data_sd7.pdf]

Effect sizes (and their standard errors) in regression analyses of the prey diversity evenness, Shannon diversity index, proportion Chironomidae pupae and ln-transformed mean weight of prey in pellets of male pond bats. Only pellets collected in periods I, III and IV were included in the analyses, because sexually active males were only caught in those periods. In the analyses we test whether males that were sexually active have different values than the ones that were not. We also test whether mature (but not sexually) males differ from immatures. The continuous explanatory variables water depth, wind speed, temperature and pellet weight were normalized (mean=0, sd=1) prior to analysis. Fitted regression models have year as a factorial random effect. In the regression analyses of evenness and the Shannon diversity index we dealt with heteroscedasticity by including an exponential variance function. The proportion of prey that were Chironomidae pupae was analysed with a logit link function. Effect sizes that are significantly different from 0 are indicated in bold.

Effect	Evenness	Shannon index	Prop. pupae	ln(Prey weight)
(Intercept)	**0.7305 (0.0527)**	**0.7419 (0.1144)**	**-2.3769 (0.3074)**	**1.0898 (0.1934)**
Sexually Active	-0.0185 (0.0530)	-0.2019 (0.1082)	0.1590 (0.3071)	-0.1280 (0.1955)
Mature vs Imma.	0.0164 (0.0444)	0.0943 (0.0913)	0.2578 (0.2612)	0.0022 (0.1649)
Water Depth	-0.0128 (0.0290)	0.0319 (0.0551)	0.1407 (0.1629)	0.0990 (0.1005)
Peat vs other soil	0.0559 (0.0521)	0.0132 (0.1012)	-0.5580 (0.2937)	0.3166 (0.1837)
Wind speed	-0.0411 (0.0252)	-0.0484 (0.0506)	-0.1664 (0.2013)	-0.0377 (0.0899)
Temperature	0.0429 (0.0246)	**0.2093 (0.0540)**	-0.1274 (0.1848)	**0.2809 (0.0942)**
Pellet weight	**-0.0588 (0.0291)**	-0.0602 (0.0509)	0.0514 (0.1355)	-0.0061 (0.0934)
